# Supplementary material for: Aging and Light Stress Result in Overlapping and Unique Gene Expression Changes in Photoreceptors
Source: Genes (Basel). 2022 Jan 29;13(2):264. doi: 10.3390/genes13020264 (PMC8872477; doi:10.3390/genes13020264)
Supplement: Supplementary file 1 [file genes-13-00264-s001.zip › Figure S1.pdf]

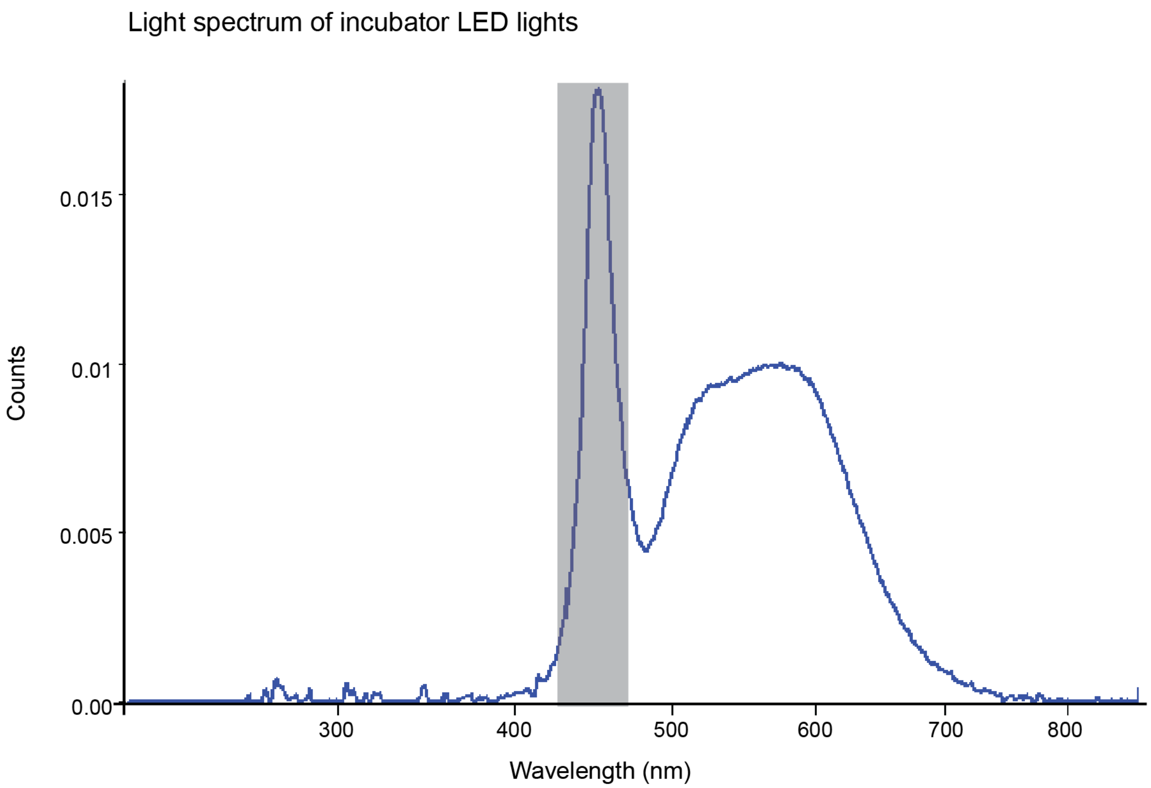

Figure S1: Light spectrum of 12:12 *Drosophila* incubator. Visible is the blue light peak, followed by the broad peak in the visible spectrum indicative of white LEDs. Grey shading represents the primary excitation wavelengths of GFP.
